# Microbial species associated with dental caries found in saliva and *in situ* after use of self-ligating and conventional brackets

**DOI:** 10.1590/1678-7757-2018-0426

**Published:** 2019-04-11

**Authors:** Ana Zilda Nazar BERGAMO, Mirian Aiko Nakane MATSUMOTO, Cássio do NASCIMENTO, Marcela Cristina Damião ANDRUCIOLI, Fábio Lourenço ROMANO, Raquel Assed Bezerra SILVA, Léa Assed Bezerra SILVA, Paulo NELSON-FILHO

**Affiliations:** 1 Universidade de São Paulo, Faculdade de Odontologia de Ribeirão Preto, Departamento de Clinica Infantil, Ribeirão Preto, São Paulo, Brasil.; 2 Universidade de São Paulo, Faculdade de Odontologia de Ribeirão Preto, Departamento de Materiais Dentários e Prótese, Ribeirão Preto, São Paulo, Brasil.

**Keywords:** Dental caries, Viridans *Streptococci Candida* ssp, Molecular biology, Orthodontic brackets

## Abstract

**Objectives:**

Enamel demineralization is among the main topics of interest in the orthodontic field. Self-ligating brackets have been regarded as advantageous in this aspect. The aim of this study was to evaluate the break homeostasis in the oral environment and the levels of microorganisms associated with dental caries among the different types of brackets.

**Material and Methods:**

Twenty patients received two self-ligating brackets: In-Ovation^®^R, SmartClip^TM^, and one conventional Gemini^TM^. Saliva was collected before bonding (S0), 30 (S1) and 60 (S2) days after bonding. One sample of each bracket was removed at 30 and 60 days for the *in situ* analysis. Checkerboard DNA-DNA Hybridization was employed to evaluate the levels of microbial species as-sociated with dental caries. Data were evaluated by nonparametric Friedman and Wilcoxon tests at 5% significance level.

**Results:**

The salivary levels of *L. casei* (p=0.033), *S. sobrinus* (p=0.011), and *S. sanguinis* (p=0.004) increased in S1. The *in situ* analyses showed alteration in *S. mutans* (p=0.047), whose highest levels were observed to the In-Ovation^®^R.

**Conclusions:**

The orthodontic appliances break the salivary homeostasis of microorganisms involved in dental caries. The contamination pattern was different between self-ligating and conventional brackets. The In-Ovation^®^R presented worse performance considering the levels of cariogenic bacterial species.

## Introduction

Enamel demineralization and cavities are among the main topics of interest in the orthodontic field.^1^ ^,^ ^2^ Dental caries are caused by the effects of acid products on the carbohydrate metabolism of bacterial species, mainly *S. mutans, S. sobrinus,* and *Lactobacillus* ssp.^3^ ^,^ ^4^ These species grow 6 or 12 weeks after orthodontic appliance bonding.^5^

Among the microbial species involved in the dental caries, mutans streptococci are the main etiological agent. In dental biofilm, which is correlated with dental caries, *Streptococcus sobrinus* and *Streptococcus mutans* are the most frequently isolated microorganisms.^6^ Recently, studies found that the coexistence of *S. mutans* and *S. sobrinus* is an important risk factor for the multi-colonized patient in the development of dental caries.^7^ ^,^ ^8^ The levels of the microorganism associated with the disruption of oral microbiota homeostasis are essential to determine caries risk and activity.

Simultaneously, the literature emphasizes the importance of *S. mitis, S. oralis, S. sanguinis, S. salivarius,* and *S. gordonii* as initial colonizers, since they provide attachment points for other species and could promote or antagonize the existence of *S. mutans* and *Lactobacillus*.^9^ ^-^ ^11^ In addition, *Candida spp.* are commonly found colonizing oral cavity with heavy infection by *S. mutans* and have been related to reduced pH levels. Previous research suggests that the interaction between these species could help develop dental caries.^12^ ^,^ ^13^

Self-ligating brackets were introduced in 1930 to reduce patient discomfort and the time spent chair-side . These brackets have progressively become part of the typical orthodontic routine. A systematic review showed that the self-ligating brackets improved oral hygiene because they retain less dental plaque and less bacterial contamination. This is a result of the design of self-ligating brackets and the absence of the elastomeric and metallic ligature. Self-ligating brackets could be divided into two categories: 1) active, which has a spring clip that presses against the archwire in the bracket slot such as In-Ovation^®^R (Dentsply – GAC), and 2) passive, in which the clip does not press against the archwire such as the SmartClip^TM^ (3M Unitek).^14^ ^-^ ^16^

Previous studies describe that different types of brackets could influence bacterial contamination.^16^ ^-^ ^19^ However, other authors have not been as concerned in assess the microbial contamination of self-ligating brackets after clinical use.^20^ ^,^ ^21^ To date, according to a recently published systematic review,^22^ the authors concluded there is insufficient evidence to support a possible influence of (conventional or self-ligating) brackets on the bacterial colonization, and they are limited to *S. mutans*. Although the microbial profile seems to be distinct from different brackets, the impact of this condition on the development of dental caries is still not conclusive.

Therefore, the aim of this randomized clinical study was to assess the profile of microbial species colonizing conventional or self-ligating brackets and saliva. Our null hypothesis is that orthodontic appliances do not break the homeostasis in the oral environment, measured by saliva, and that there are no significant differences in the bacterial levels among the different types of brackets on *in situ* analysis.

## Material and methods

This protocol research was approved by the Institutional Research Ethics Committee (Process #0062.0.138.000-10).

Twenty patients (11 men and 9 women; mean age=13.3±1.03 years) from the Orthodontics Clinic of our Institution were selected after a screening examination, which included a full medical and dental history and an intra-oral examination. Patients were not included in the study if they: (i) had previous orthodontic treatment; (ii) systemic disorders; (iii) used antibiotics in the three months preceding the study; (iv) received periodontal treatment three months preceding the study; (v) had a smoking habit (or if they were former smokers); (vi) craniofacial anomalies; (vii) severe tooth crowding, overjet, and overbite; (viii) mouth breathing.

Schematic drawings of the six anterior teeth were designed to randomly distribute the different types of brackets into the six teeth previously selected for bonding. The brackets were numbered from 1 to 6 in the following distribution: number 1 matched the In-Ovation^®^R bracket, number 2 matched the SmartClip^™^ bracket, and number 3 matched the Gemini^™^ bracket removed 30 days after bonding (first dental set analyzed); number 4 matched the In-Ovation^®^R bracket, number 5 matched the SmartClip^™^ bracket, and number 6 matched the Gemini^™^ bracket removed 60 days after bonding (second dental set analyzed). A total of 120 brackets were investigated in this study: In-Ovation^®^R (n=20), SmartClip^™^ (n=20), and Gemini^™^ (n=20) 30 days after bonding; In-Ovation^®^R (n=20), SmartClip^™^ (n=20), and Gemini^™^ (n=20) 60 days after bonding.

This random assignment also ensured that the number of brackets removed 30 or 60 days after bonding was similar for each anterior tooth analyzed on both left and right sides.

**Figure 1:**
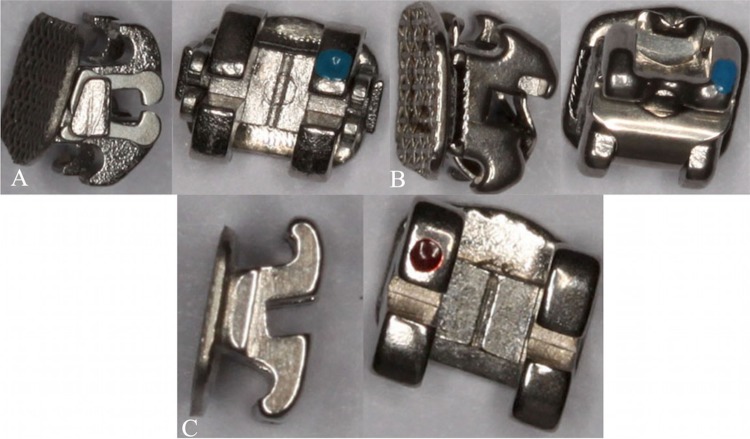
Bracket design. A- Self-ligating bracket SmartClipTM; B- Self-ligating bracket In-Ovation®R; C- Conventional bracket GeminiTM

The brackets were bonded to the upper incisor and canine teeth. Two In-Ovation^®^R (Dentsply, GAC – Islandia, NY, USA), two SmartClip^TM^ (3M Unitek, Monrovia, CA, USA), and two conventional brackets: Gemini^TM^, (3M Unitek, Monrovia, CA, USA) associated with elastomeric ligatures were bonded. Transbond Etching Primer (3M Unitek, Monrovia, Calif, USA) was applied with a microbrush, and Transbond XT (3M Unitek, Monrovia, Calif, USA) composite was utilized to the bracket. The orthodontic archwire 0.014” was placed in a passive configuration. Figure 1 illustrates the design of the three different brackets.

As previously described by Bergamo, et al.^23^ (2016), plaque index and gingival bleeding index were measured using a PCPUNC-BR15 probe (HuFriedy from Brazil, Rio de Janeiro, RJ, Brazil), and initial values (median, 1^st^ first quartile, and 3^rd^ third quartile) to the first (30 days after bonding) and second (60 days after bonding) dental set analyzed were 1.0 (1.0-2.0). The gingival bleeding index of 1.0 (1.0-2.0) indicated the health of the sample. According with Bergamo, et al.^23^ (2016), only the plaque index increased significantly 60 days after bonding of SmartClip^TM^ Brackets.

Instruction on hygiene was performed by one operator: the modified Bass technique, three times a day. The patients received the same dental tooth brush (Professional^®^, Colgate-Palmolive Indústria e Comércio Ltda, São Bernardo do Campo, SP, Brazil) and toothpaste (Oral-B^®^ Pro-Saúde^©^, 2012 Procter & Gamble of Brazil).

Each debonding bracket was transferred to 150 µL of TE (10 mm Tris-HCl, 1 mm EDTA, pH 7.6), centrifuged in Mixtron and then removed by sterilized pliers, followed by the addition of 100 µL of 0.5 M NaOH, and stored at -20˚C until checkerboard DNA–DNA hybridization processing according to Bergamo, et al.^24^ ^,^ ^25^ (2017, 2018).

Before bonding, 1 mL of non-stimulated saliva was collected. After 30 seconds of centrifugation, 30 µL was transferred to Eppendorf tubes (Eppendorf AG Barkhausenweg 1 22339 – Hamburg, Germany) with a content of 120 µL of buffer solution [10 mM Tris-HCL (Sigma-Aldrich, Co., St. Louis, MO, USA)], pH 7.6. Then 100 µL of NaOH (Labsynth) was added to the Eppendorf tubes. They were stored at -20°C until checkerboard DNA–DNA hybridization. After 30 and 60 days, new saliva sample preparations were carried out.

### Checkerboard DNA-DNA hybridization

After thawing, the samples were boiled for 5 min. After cooling, 800 µL of 5 M ammonium acetate was added to each tube, and the contents of the tube were applied to the extended slot in the MiniSlot apparatus (Immunetics Inc., Boston, MA, USA), concentrated onto a 15x15 cm nylon membrane (Hybond Nþ, Amershan Biosciences, Buckinghamshire, UK), and baked for 2 h at 80°C. Control samples defined amounts of genomic DNA corresponding to either 10^5^ or 10^6^ of the following bacterial cells: *Streptococcus mutans* (ATCC-25175), *Streptococcus sobrinus* (ATCC-27352), *Streptococcus gordonii* (ATCC-10558), *Streptococcus mitis* (ATCC-49456), *Streptococcus oralis* (ATCC-35037), *Streptococcus sanguinis* (ATCC-10556), *Lactobacillus casei* (ATCC-393), *Candida tropicalis* (ATCC-13803), *Candida krusei* (ATCC-2159), *Candida glabrata* (ATCC-66032), *Candida dubliniensis* (ATCC-44508), and *Candida albicans* (ATCC-10231) was applied to two control slots.

The membranes were pre-hybridized in buffer hybridization [NaCl 0.5 M; blocking reagent 0.4% (w/v)]. Then the membranes were placed in a Miniblotter 45 (Immunetics, USA). Fluorescein-labeled genomic probes were diluted in 150 ml of hybridization solution, applied in the individual lanes of the Miniblotter, and this apparatus was placed in a sealed plastic bag containing sheets of wetted paper towel. Under gentle agitation, hybridization was performed overnight at 60°C. The following day, the membranes were washed twice in a solution of 2 M urea, 0.1% sodium dodecyl sulfate (SDS), 50 mM NaH_2_PO_4_ pH 7.0, 150 mM NaCl, 1 mM MgCl_2_, and 0.2 blocking reagent at 65°C for 30 min, and twice in a solution of 1 M Tris base, 2 M NaCl, and 1 M MgCl_2_ for 15 min at room temperature.

The hybrids were detected by chemiluminescence using the Gene Images CDP-Star detection module (GE healthcare). The membrane was exposed to ECL Hyperfilm MP (GE healthcare) for 10 min, and chemiluminescent signals were detected. The image was digitized and analyzed by the TotalLab™ Quant v13 software (TotalLab Ltd, Newcastle, UK). The number of microorganisms colonizing each site could be expressed in terms of levels (μg).

### Statistical analyses

The significant differences among the three periods evaluated in saliva were determined by nonparametric Friedman’s test and Dunn’s *post hoc*, since data did not fit model assumptions (data were shown right and left-skewed).

In the same way, the statistically significant differences among the three brackets, *in situ* evaluation, were carried out. Wilcoxon’s test was used to determine the differences between the microbial levels at 30 and 60 days after bonding.

Differences were considered significant when p<0.05. The SPSS 21.0.0 statistical software (SPSS Inc., Chicago, IL, USA) was used for data analysis.

## Results

**Figure 2:**
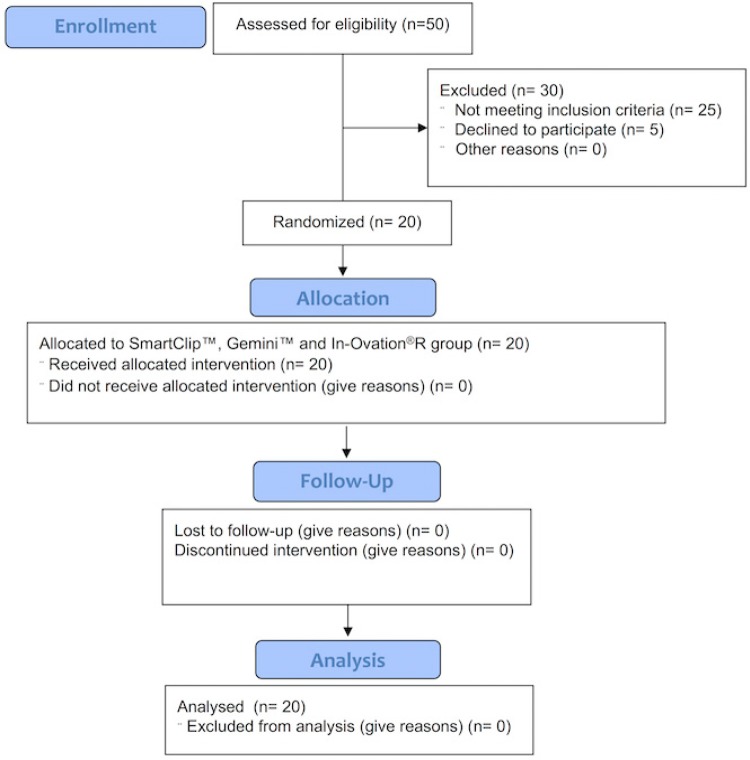
Flow diagram of allocated intervention and follow-up

A total of fifty participants were recruited. Thirty of them were excluded, and twenty people were enrolled in this study. Figure 2 highlights the flowchart of participants through the trial.

**Figure 3:**
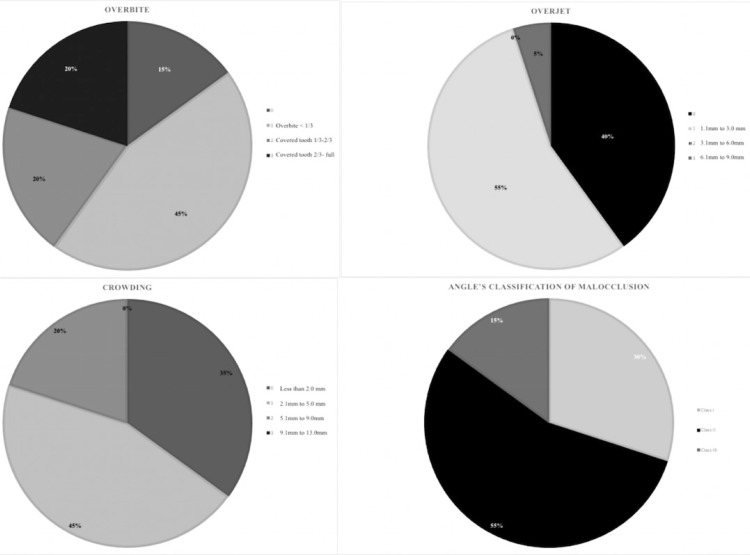
Demographic characteristics and malocclusion features of the sample

Figure 3 shows all demographic characteristics and malocclusion features of the included subjects.

Before bonding, the presence of all microbial species in the saliva was verified, except for *S. gordonii.* The levels of *C. krusei* decreased in 30 (p=0.027) and 60 (p=0.00001) days after bonding when compared with levels before bonding. *C. glabrata* showed highest levels before bonding. Significant difference was observed for *S. oralis*, whose levels decreased 60 days after bonding when compared with S0 (p=0.48) and S1 (p=0.40). An increased level of *S. sobrinus* (p=0.011) and *L. casei* (p=0.033) was observed 30 days after bonding when compared with levels before bonding. *S. sanguinis*, whose levels in S1 were higher than S0 (p=0.004) and S2 (p=0.004) (Table 1).

**Table 1:** Microbial count (µg x 105) in the saliva

Microbial Species		S0	S1	S2	p (Friedman)
*S. sobrinus*	(Q1-Q3)	0.00-2.71	2.67-3.25	0.00-3.30	0.024*
	Median	0	2.8	0	
*L. casei*	(Q1-Q3)	0.00-3.25	0.00-2.14	0.00-0.00	0.006*
	Median	0	1.7	0	
*S. mutans*	(Q1-Q3)	0.00-0.00	0.00-1.93	0.00-1.72	0.64
	Median	0	0	0	
*S. gordonii*	(Q1-Q3)	0.00-0.00	0.00-0.00	0.00-0.00	0.15
	Median	0	0	0	
*S. mitis*	(Q1-Q3)	0.00-0.00	0.00-1.88	0.00-1.97	0.15
	Median	0	0.8	0	
*S. oralis*	(Q1-Q3)	0.00-2.74	0.00-2.52	0.00-0.00	0.018*
	Median	0	2.09	0	
*S. sanguinis*	(Q1-Q3)	0.00-2.73	2.02-2.86	0.00-2.40	0.008*
	Median	0	2.49	2.01	
*C. tropicalis*	(Q1-Q3)	0.00-2.71	0.00-1.88	1.67-2.05	0.37
	Median	2.6	1.61	1.97	
*C. krusei*	(Q1-Q3)	2.60-2.90	0.40-2.63	0.00-2.18	0.0001*
	Median	2.72	2.28	1.92	
*C. glabrata*	(Q1-Q3)	0.00-2.69	0.00-0.00	0.00-0.00	0.049*
	Median	1.28	0	0	
*C. dubliniensis*	(Q1-Q3)	0.00-2.74	0.00-2.74	0.00-2.16	0.43
	Median	1.31	2.15	0	
*C. albicans*	(Q1-Q3)	0.00-2.77	0.00-1.87	0.00-2.23	0.99
	Median	0	0.66	2.1	

S0: saliva sample before bonding; S1: saliva sample 30 days after bonding; S2: saliva sample 60 days after bonding; * Friedman statistically significant difference; M: median; Q1: fist quartile; Q3: third quartile

The *in situ* analysis showed the presence of all species in all brackets. Table 2 and Table 3 show the microbial levels, in the different periods of this study.

**Table 2:** Microbial count (µg x 105) *in situ* sample

Bacterial species	Time	SmartClip^**TM**^	Gemini^**TM**^	In-Ovation^**®**^R	p
		Median	Median	Median	
		(Q1-Q3)	(Q1-Q3)	(Q1-Q3)	
*S. gordonii*	T1	0.63	0	2.05	0.25
		(0.00-2.68)	(0.00-3.13)	(0.00-2.86)	
	T2	1.63	1.1	0.94	0.37
		(0.00-2.79)	(0.00-2.81)	(0.00-2.75)	
*S. mitis*	T1	2.25	2.5	2.63	0.23
		(0.00-2.84)	(0.00-2.94)	(0.00-3.61)	
	T2	2.22	2.61	2.46	0.43
		(1.68-3.31)	(1.15-2.92)	(1.78-3.38)	
*S. oralis*	T1	0	1.5	0	0.35
		(0.00-3.08)	(0.00-2.98)	(0.00-3.02)	
	T2	2.61	2.07	2.57	0.77
		(1.42-2.71)	(1.72-2.68)	(1.57-2.74)	
*S. sanguinis*	T1	0	0	0.67	0.56
		(0.00-2.13)	(0.00-2.11)	(0.00-2.22)	
	T2	2.64	2.59	2.43	0.83
		(0.38-3.18)	(0.0-2.96)	(00.0-3.12)	
*S. sobrinus*	T1	2.29	1.03	2.69	0.61
		(0.00-3.31)	(0.00-3.24)	(0.00-3.38)	
	T2	2.45	2.28	2.66	0.87
		(1.45-2.97)	(0.00-3.17)	(0.32-3.12)	
*L. casei*	T1	1.88	0.83	2.38	0.31
		(0.00-2.71)	(0.00-2.71)	(0.00-3.12)	
	T2	0	0	1.89	0.36
		(0.00-2.45)	(0.00-2.45)	(0.00-2.49)	
*S. mutans*	T1	0	1.03	2.66	0.087
		(0.00-2.91)	(0.00-2.99)	(0.00-3.22)	
	T2	1.17	1.69	1.9	0.047*
		(0.00-2.48)	(0.00-2.48)	(0.25-2.67)	

T1: 30 days after bonding; T2: 60 days after bonding; M: median; Q1: fist quartile; Q3: third quartile; *Statistically significant difference Firedman test

**Table 3:** *Candidas* ssp count (mgx105) *in situ* sample

*Candidas* ssp	Time	SmartClip^**TM**^	Gemini^**TM**^	In-Ovation^**®**^R	p
		Median (Q1-Q3)	Median (Q1-Q3)	Median (Q1-Q3)	
*C. tropicalis*	T1	2.14	2.07	2.49	0.89
		(0.00-2.91)	(0.00-3.08)	(0.47-3.21)	
	T2	2.66	2.6	2.67	0.52
		(2.18-2.80)	(2.04-2.71)	(2.09-2.80)	
*C. krusei*	T1	2.29	0	1.97	0.62
		(0.00-3.06)	(0.00-2.62)	(0.00-2.94)	
	T2	1.94	2.31	2.47	0.098
		(0.00-2.71)	(0.00-2.68)	(0.00-2.76)	
*C. glabrata*	T1	2.33	2.03	2.34	0.72
		(0.00-2.90)	(0.00-2.63)	(0.00-2.99)	
	T2	2.64	2.32	2.38	0.43
		(2.10-2.73)	(1.44-2.69)	(0.25-2.81)	
*C. dubliniensis*	T1	2.24	2.5	2.65	0.56
		(0.00-3.34)	(0.00-3.30)	(0.00-3.28)	
	T2	2.13	2.38	1.57	0.63
		(0.46-2.65)	(0.00-2.66)	(00.0-2.63)	
*C. albicans*	T1	0	0.99	0	0.4
		(0.00-2.82)	(0.00-2.69)	(0.00-2.43)	
	T2	0	0	0	0.5
		(0.00-1.43)	(0.00-2.57)	(0.00-2.60)	

T1: 30 days after bonding; T2: 60 days after bonding; M: median; Q1: fist quartile; Q3: third quartile; *Statistically significant difference Firedman test

No significant difference was observed in the contamination levels of *Candida ssp.* among different brackets by the Friedman test (Table 3).

The bacterial levels showed a significant difference for the *S. mutans* 60 days after bonding among the three different brackets, by Friedman test. The highest levels were observed in the In-Ovation^®^R.

Although a statistically significant difference was not found, the highest levels of *L. casei* and *S. sobrinus* were also observed in the In-Ovation^®^R (Table 2).

When the *in situ* levels of microbial species were compared 30 or 60 days after bonding, a significant difference occurred in the *S. sanguinis*, which increased levels for all types of brackets. Table 4 shows the p-value.

**Table 4:** Wilcoxon test for microbial levels *in situ* analysis

Microbial species	SmartClip^**TM**^	Gemini^**TM**^	In-Ovation^**®**^R
	p Wilcoxon test	p Wilcoxon test	p Wilcoxon test
*S. gordonii*	0.35	0.52	0.58
*S. mitis*	0.23	0.76	0.6
*S. oralis*	0.21	0.18	0.09
*S. sanguinis*	0.003*	0.044*	0.035*
*S. sobrinus*	0.41	0.72	0.85
*L. casei*	0.25	0.47	0.35
*S. mutans*	0.8	0.43	0.95
*C. tropicalis*	0.11	0.17	0.69
*C. krusei*	0.44	0.19	0.74
*C. glabrata*	0.5	0.29	0.66
*C. dubliniensis*	0.91	0.81	0.099
*C. albicans*	0.13	0.26	0.92

Comparison between 30 and 60 days after bonding; *Statistically significant difference

## Discussion

In this study, we investigated through the Checkerboard DNA-DNA hybridization analysis, the microbial colonization of conventional or self-ligating brackets and the levels of microorganisms recovered from saliva of healthy individuals. Moderate to high levels of pathogens were found in both conventional and self-ligating brackets, and in the saliva. An increase in the *S. Sobrinus*, *L. casei,* and *S. sanguinis* at S2 confirmed the disruption of the homeostasis in the oral environment promoted by the orthodontic appliances. The *in situ* analysis allowed a distinct pattern in microbial adhesion in the different bracket designs for the *S. mutans* (the highest level was observed in the In-Ovation^®^-R brackets), and an increase in levels of the *S. sanguinis* (comparing T1 and T2). No significant differences were recorded for fungal levels over time *in situ* analyses.

This fact increased the saliva levels of *S. sobrinus* over time and, its coexistence with *S. mutans* and *L. casei* indicated a high microbial caries risk in this experimental population, which evaluated the early stages of orthodontic treatment. According to several studies, high counts of salivary bacterial species (*S mutans, L. casei* and *S. sobrinus*) imply risk of caries.^3^ ^,^ ^26^ Saliva may influence the formation of the acquired pellicle, determining which microorganisms are able to attach and colonize dental surfaces.^27^ ^,^ ^28^ We must emphasize that *L casei* is associated with deep carious lesions, thus it has no capacity for adhesion or the ability to be maintained mechanically.^9^ ^,^ ^10^ Their levels could not remain high for long periods in saliva. *S. sobrinus* is a highly cariogenic species. It presents high acidogenicity, synthesizes extracellular polysaccharides from sucrose, promotes attachment points, has capacity to store compounds that could be converted into acid during periods when sugars are not available, and it grows in a low pH environment.^7^ ^,^ ^8^

The *in situ* analysis of this study showed a different contamination pattern when compared with salivary analysis. While the salivary analysis showed that *S. sobrinus*, *L. casei* and *S. sanguinis* increased over time, the *in situ* analysis allowed a distinct pattern in microbial adhesion in the different bracket designs for *S. mutans* and an increase in levels of *S. sanguinis*. The main stream of the studies, which evaluated different contamination patterns between self-ligating and conventional brackets, analyzed the saliva of different patients. Previous studies on the epidemiology of dental caries and periodontal disease indicated that each person presents a singular risk factor of these diseases, since they are multifactorial, and different contamination patterns are identified.^29^ ^-^ ^31^

When we compared the relative effect of time factor (T1 and T2) for all brackets, we found an increase in the *S. sanguinis* levels. *S. mutans* showed a significant difference among the three brackets, with the highest value assigned to the self-ligating In-Ovation^®^R. However, it did not show an increase when T1 was compared with T2, and in individual brackets analysis this fact could be associated with the interaction of antagonist species. The null hypothesis that the different types of brackets did not affect the microbial levels was rejected.

The relationship among *S. sanguinis*, *S. gordonii*, *S. oralis* and *S. mitis* promote the congregation of possible attachment of new species to the tooth surface. *S. sanguinis* are a regular member of the dental plaque and are considered to be a beneficial bacterial species concerning dental caries, since it is an antagonist to *S. mutans*. This occurs due to its inhibitory substance production, such as mutacin and hydrogen peroxide (H_2_O_2_).^9^ ^,^ ^11^ ^,^ ^32^

The alteration of fungal levels over time was expected, since our experimental population consisted of healthy young participants who are not subject to the proliferation of opportunist microorganisms such as *Candida spp*. Conversely, recent studies have shown an interaction between *S. mutans* and *Candida spp.* ^12^ ^,^ ^13^

Some species have not been identified or showed lower signals. This could be attributed in part to the small amount of biofilm collected. Also, species found below the threshold of 10^4^ cells result in non-detectable hybridization signals. The possibility of nonspecific binding is another point between the proportion of bacterial DNA and other macromolecules. Cross-reactions may occur if the probes are employed to detect species over 10^7^ range. Moreover, the amount of NaOH used in the buffering could not lyse large DNA samples. These facts may have influenced the results of this study. Additional studies are necessary to accurately elucidate the changes analyzed by this method of molecular biology.

In this article, we focused only on the microbial ecology of different brackets. Dental caries is an endogenous disease, caused by change of mutualistic symbiosis in the microbial ecosystem, associated with local environmental changes, sugar and carbohydrate intake, salivary secretion, and previous dental caries history. Some diseases such as mouth breathing syndrome increased susceptibility to dental caries and other oral infections^33^ ^,^ ^34^ in order to minimize the bias, the sample in this study was not composed of mouth-breathing patients. Considering these aspects, future microbiological studies should focus on all these aspects to better understand the physiological mechanisms that maintain the dynamic stability in dental biofilms and dental caries in orthodontic treatments, as well as the impact on oral health.

## Conclusion

Within the limitations of this study, we can conclude that orthodontic appliances disrupted the homeostasis of microorganisms commonly involved in dental caries. The type of bracket may influence the bacterial adhesion, since a significant difference was found for the *S. mutans* levels among the three brackets over time, with the highest value observed for the self-ligating bracket In-Ovation^®^R. A similar pattern of colonization was observed for *S. sobrinus* and *L. casei,* whose highest value was detected in the In-Ovation^®^R bracket as well.
